# 

**Published:** 1895-12-07

**Authors:** 


					jxijj; iioui  AB^OJ V-TF!,v pu RKi theiefcorft BESTe xfecjimbek /th, i?yo.
CADBURVS iof w. CADBURY'S
COCOA HT JhI COCOA
C?^ "The standard of highest purity at present Jjb NO ALKALIES 11
i,io " t- j
attainable."?LaneeU
NO ALKALIES USED.
V S.
'RWICH HOSPLTAl
No. 480. Vol. XIX.
Saturday,
Dec. 7th, 1895.
WITH EXTRA NURSING SUPPLEMENT.
[Registered at the General Post Office as a Newspaper for
transmission in the Unite! Kingdom and Abroad.]
PRICE TWOPENCE.
Per Annum, 10s. 6d., post free.
Monthly Parts, 6d.; post free, 8d.
Ditto per Annum, 8s.6d., post free.

				

